# Geometry, reactivity descriptors, light harvesting efficiency, molecular radii, diffusion coefficient, and oxidation potential of RE(I)(CO)_3_Cl(TPA-2, 2′-bipyridine) in DSSC application: DFT/TDDFT study

**DOI:** 10.1186/s13065-024-01218-y

**Published:** 2024-06-10

**Authors:** Dereje Fedasa Tegegn, Habtamu Zewude Belachew, Shuma Fayera Wirtu, Ayodeji Olalekan Salau

**Affiliations:** 1 Department of Chemistry, College of Natural and Computational Science, Dambi Dollo University, P. O. Box. 260, Dambi Dollo, Oromia Ethiopia; 2 Department of Electrical/Electronics and Computer Engineering, Afe Babalola University, Ado-Ekiti, Nigeria; 3 Saveetha School of Engineering, Saveetha Institute of Medical and Technical Sciences, Chennai, Tamil Nadu India

**Keywords:** Dye-Sensitized solar cells, Light harvesting efficiency, Molecular radii, Diffusion coefficient, Excited oxidation potential

## Abstract

Dye-sensitized solar cells (DSSCs) are an excellent alternative solar cell technology that is cost-effective and environmentally friendly. The geometry, reactivity descriptors, light-harvesting efficiency, molecular radii, diffusion coefficient, and excited oxidation state potential of the proposed complex were investigated. The calculations in this study were performed using DFT/TDDFT method with B3LYP functional employed on the Gaussian 09 software package. The calculations were used the 6–311 +  + G(d, p) basis set for the C, H, N, O, Cl atoms and the LANL2DZ basis set for the Re atom, with the B3LYP functional.. The balance of hole and electron in this complex has increased the efficiency and lifetime of DSSCs for photovoltaic cell applications. The investigated compound shows that the addition of the TPA substituent marginally changes the geometric structures of the 2, 2′-bipyridine ligand in the T_1_ state. As EDsubstituents were added to the compound, the energy gap widened and moved from E_LUMO_ (− 2.904 eV) (substituted TPA) to E_LUMO_ (− 3.122 eV) (unsubstituted). In the studying of solvent affects; when the polarity of the solvent decreases, red shifts appears in the lowest energy an absorption and emission band. Good light-harvesting efficiency, molecular radii, diffusion coefficient, excited state oxidation potential, emission quantum yield, and DSSC reorganization energy, the complex is well suited for use as an emitter in dye-sensitized solar cells. Among the investigated complexes mentioned in literature, the proposed complex was a suitable candidate for phosphorescent DSSC.

## Introduction

The core of dye-sensitized solar cells based on solar radiation is the concept of charge distribution at the point of interaction of two materials with different electron movement processes [1]. Unlike a standard semiconductor that performs both functions, the device constitutes a stage at which the transport of light absorption and charge carrier transport can be isolated [2]. As a result, DSSCs provide a more practical and financially viable alternative to current p–n junction solar systems. In addition to solid-state devices, dye-sensitized solar cells (DSSCs) are an excellent alternative solar cell technology with cost-effective and environmentally friendly properties [3]. In a conventional DSSC, light is trapped by a sensitizer (dye) grafted onto the surface of a thin TiO_2_ semiconductor film. Charge separation at the sensitizer-TiO_2_ interface is caused by the photoinduced movement of electrons from the dye to the conduction band (CB) of the semiconductor. The charge collectors serve to transport the created electron–hole pair to the external circuit. A redox pair structure (often a natural compound such as an iodide/triiodide pair) regenerates the colored particle while it is regenerated by electrons at the counter terminal.

Regardless, in order to work on DSSC exhibition, it is important to explore creative materials such as host materials [4, 5]. Since the presence of metal complexes exhibits a strong SOC that significantly accelerates the single-to-triplet intercalation (ISC), we used a third series of d^6^-mediated metal complexes with suitable organic ligands. The creation of highly efficient optical compounds requires the use of organic ligands that allow various electronic transitions between unique energy levels associated with metal atoms [6–8]. The bidentate heteroaromatic $\widehat{\text{NN}}$ ligand complexes with d^6^ 3rd row transition metal ions such as Re(I), Ru(II), and Os(II) exhibit remarkable photophysical properties. Rhenium-containing complexes with 2,2′-bipyridine typically exhibit robust, enduring iridescence. 2,2′-bipyridine is a bidentate ligand with strong interaction for the Re(I). It is easy to change it by adding different groups of substituents at different places. To change the energy level of the 2, 2′-bipyridine ligand and to construct highly efficient DSSCs, it is advantageous to use electron-donating groups such as the TPA substituent [9, 10]. The low luminescence efficiency and intrinsic quantum efficiency, on the other hand, are produced by unequal charge carrier for electrons and openings in the discharge layer of the DSSC system. Because these unsubstituted compounds have good electron transfer abilities but poor hole transfers properties [11]. The authors attempted to solve the problem of low light harvesting efficiency, intrinsic quantum efficiency, and luminescence performance of unsubstituted complexes inside the DSSC gadget by means of a theoretical treatment of the electronic structure design and photophysical characteristics of TPA-substituted of proposed complex.

## Methods

### Proposed computational methods

The geometries of the singlet ground state (S_0_) and the lowest-lying excited triplet state (T_1_) of the investigated compound were optimized in the gas phase using the DFT technique [12]. In addition to the 6–311 +  + G(d, p) basis set for C, H, N, O and Cl atoms, the B3LYP exchange correlation functional [13] can also accurately evaluate the LANL2DZ basis set with double ζ quality for the Re atom. LANL2DZ for Re and 6–311 +  + G(d, p) for the premise set of different molecules are also remembered for a complementary contribution within the Gaussian arrangement of the calculation [14]. Vibration frequency was conducted to ensure that the improved structures were undoubtedly stable structures. Accordingly, they are the smallest points on the potential energy surface with no imaginary frequency for any design. Using the optimal structures, the energy level and contour plot of the HOMO and LUMO of the studied complex were obtained.

Charged state calculations were investigated using the TDDFT approach with respect to a simplified construction of the investigated complex with indistinguishable functional and basis sets [15, 16]. The absorption and emission spectra of the complex were estimated using the TDDFT method on the optimized S_0_ and T_1_ structures. GAMESS software was used to model the absorption spectra of the studied compound to obtain the best spectra. PCM is used in the TDDFT calculation to account for the impact of the solute around the particle. Electron density plots for FMO were generated using Gaussian software. The involvement of positive and negative ions in the production of “electron holes” is key to their use as DSSC materials. Subsequently, the + ve and −ve energy states of the unbiased atom were compared to calculate ionization potentials (IPs), electron affinities (EAs) and reorganization energies. Descriptors of complex reactivity, light harvesting efficiency, molecular radii, diffusion coefficient and excited oxidation potential were calculated using HOMO and LUMO energies. All calculations were performed using the software application Gaussian 09 [17].

## Results and discussion

### Stable geometries of complex

**Fig. 1:**
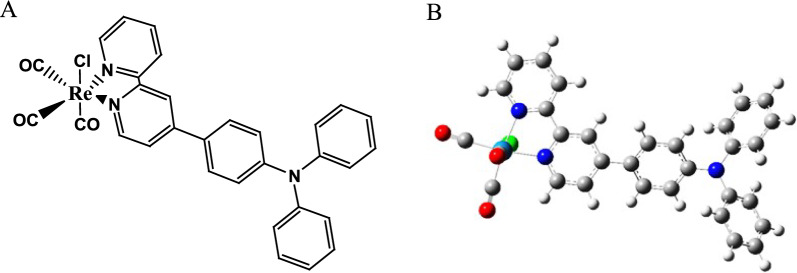
Complex chemical structure (**A**) and optimized geometry (**B**)

The explored complex chemical structure and optimized ground state geometry were demonstrated (Fig. 1). Table 1 accumulates exploratory qualities for complex in view of crystallographic information from the previous reported [18], as well as the examined complex's chosen bond lengths and bond angles in the optimal ground state (S_0_) and lowest lying triplet state (T_1_). The geometry is formed by the substituted TPA on the bidentate ligand, CO, and Cl atom around the Re(I) atom. The constancy of the complex's ideal geometries was verified using frequency analyses that reveal that there is no imaginary frequency for any configuration. Figure 1 shows that this complexes via TPA have a similar face octahedral coordination with the bidentate ligand, CO, and Cl around the Re atom. Complexes display normal Re(I) tricarbonyl diamine complex properties in terms of bond lengths and bond angles, as shown in Table 1.

**Table 1:** the computed parameters of complex in S_0_ & T_1_ states at the B3LYP/6–311 +  + G(d, p)/LANL2DZ level. Angles measured (degrees) & distances (angstroms)

	(-H) (-TPA)		
	Exptl.	S_0_	T_1_
Bond length (Å)			
Re-Cl8	2.574	2.551	2.513
Re-C2	1.915	1.909	1.933
Re-C3	1.907	1.924	1.934
Re-C4	2.058	1.925	1.970
Re-N26	2.163	2.172	2.156
Re-N27	2.171	2.167	2.086
Bond angles (^o^)			
N26-Re-N27	74.70	75.32	77.27
C2-Re-C4	95.90	91.79	89.91
C2-Re-C3	90.30	91.79	92.21
C2-Re-N26	90.70	93.88	93.27
C2-Re-N27	86.00	93.60	91.45
C3-Re-N27	97.50	75.32	77.27
C4-Re-N26	97.80	91.79	89.91
C4-Re-Cl8	–	91.79	92.21

Calculated the experimental values obtained from the crystallographic data published in the literature [18] are in good agreement. It provides strong evidence for the correctness of the theoretical approach. Small differences are observed due to the effects that the theoretical calculations do not take into account in the tightly closed and chemical environment. The study found that EWG caused a red shift in the lowest energy absorption and emission bands, while EDG caused a blue shift, finding can serve as a benchmark to compare the effects of the TPA ligand in this complex [19]. Although the close-packed lattice gives practical results, the theoretical calculations are valid for the gas phase. Substitution of TPA on the 2,2′-bipyridine ligand results in a small modification of the bond, as seen in Table 1. For the investigated compounds, the typical angle of approximately 90° between the three CO ligands in fac-Re(CO)^3+^ is unity.

In each complex, the axial Re-C bond distance is shorter than the equatorial Re-C bond distance. This is due to the axial CO opposite the Cl atom having a distinct ligand to metal back bonding capacity. The complex's estimated geometrical parameters for the T_1_ included in Table 1 and reveals geometric structures of the 2, 2′-bipyridine ligand in the T_1_ state are minimally affected by the addition of a TPA substituent. However, there are significant changes in the bond lengths and bond angles of the complex in the T_1_ and S_0_ states. The bond lengths of Re–N and Re–Cl are particularly shortened, whereas those of Re-C are lengthened. While Re(I) interactions with three CO ligands are weaker in the T_1_ state, those with the 2, 2′-bipyridine ligand are greater. As a result, the 2, 2′-bipyridine ligand has a stronger effect on the FMOs of these complexes in the T_1_ state. The varied strengths of Re(I) and TPA-2,2′-bipyridine ligands or CO ligands will result in different electron transition characteristics.

Experimental results were taken from the literature [18]. The calculated optimal parameters suggest an octahedral coordination.

### Molecular orbital properties and global reactivity descriptors

**Fig. 2:**
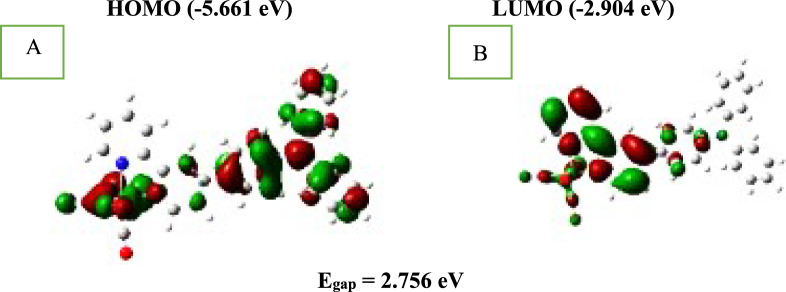
Contour plot of HOMO (**A**) and LUMO (**B**) of studied complexes

The frontal molecular orbital (FMO) properties of DSSC materials have a substantial effect on their energized states and electronic changes. FMOs, especially HOMOs and LUMOs, are related to the optical properties of the complexes. Contour plots of the HOMO (H) and LUMO (L) energy levels in the complex, as well as the principal FMO energy levels, are shown in Fig. 2. As can be seen, the studied complex's HOMOs are predominantly made up of the d(Re), p(Cl), and orbitals of CO ligands, while the LUMOs are primarily made up of the TPA-2, 2′-bipyridine ligand’s π* anti-bonding orbitals. The addition of TPA substituent groups to the 2, 2′-bipyridine ligand had no effect on the FMO compositions. When EDG groups (TPA) are introduced, the HOMOs rarely change (Fig. 2). When different substituent bunches is joined to the 2, 2′-bipyridine ligand, the energy levels LUMOs vary significantly. The introduction of EDGs (-TPA) increases E_LUMO_. As electron-donor substituent groups are added, the energy gap of the molecule widens, moving from E_LUMO_ (− 2.904 eV) (substituted by TPA) to E_LUMO_ (− 3.122 eV) (unsubstituted). Contour plot of HOMO and LUMO of studied complexes was shown in Fig. 2.

Furthermore, the quantum chemical parameters HOMO and LUMO are essential for predicting the reactivity of the substance under investigation. Descriptors of chemical reactivity that are important are studied using them, such as ionization potentials (IP), electron affinity (EA), electronegativity (EN), chemical hardness (η), chemical potential (μ), chemical softness (S), electrophilicity index (ω), electron accepting capability (ω^+^), electron donating capability (ω^−^), Nucleophilicity index (N), additional electronic charge (N_max_), and optical softness (σ^o^) are some of the terms used to describe the properties of a material [20, 21]. The energy of the HOMOs and LUMOs with all global reactivity descriptors of the studied complex was determined using the DFT technique at the B3LYP/6–311G +  + (d, p) basis set and is shown in Table 2.

$$\begin{gathered} |\Delta E| \, = {\text{E}}_{{{\text{LUMO}}}} - {\text{E}}_{{{\text{HOMO}}}} ,{\text{IP}} = \, - {\text{E}}_{{{\text{HOMO}}}} ,{\text{ EA}} = \, - {\text{E}}_{{{\text{LUMO}}}} ,EN = \frac{{\left( {I + A} \right)}}{2},\,\eta = \frac{{\left( {I - A} \right)}}{2},\mu = \frac{{\left( {I + A} \right)}}{2}, \hfill \\ S = \frac{1}{{\left( {2\eta } \right)}},\,\omega = \frac{{\mu^{2} }}{{\left( {2\eta } \right)}},\,\omega^{ + } = \frac{{\left( {I + 3A} \right)^{2} }}{{16\left( {I - A} \right)}},\,\omega^{ - } = \frac{{\left( {3I + A} \right)^{2} }}{{16\left( {I - A} \right) }}, N \, = \frac{1}{\omega },\,\Delta {\text{N}}_{{{\text{max}}}} = \frac{ - \mu }{{\eta }},and \sigma^{{\text{o}}} = \frac{1}{\Delta E } \hfill \\ \hfill \\ \end{gathered}$$

**Table 2:** Calculated energy and chemical reactivity descriptors of studied complex

S.No	Physical parameters	Values
1	E_HOMO_ (eV)	− 5.661
2	E_LUMO_ (eV)	− 2.904
3	E_Gap_ (eV)	2.756
4	Ionization potentials, IP (eV)	5.661
5	Electron affinity, EA (eV)	2.904
6	Electronegativity, EN (eV)	4.282
7	Chemical hardness, *η* (eV)	1.378
8	Chemical potential, *μ* (eV)	− 4.282
9	Chemical softness, *S* (1/eV)	0.362
10	Electrophilicity index, *ω* (eV)	6.652
11	Electron accepting capability (*ω*^+^)	4.683
12	Electron donating capability (*ω*^*−*^)	8.965
13	Nucleophilicity index (N)	0.150
14	Additional electronic charge (∆N_max_)	3.107
15	Optical softness (σ^0^)	0.362

According to the data, Egap is 2.756 eV, the smallest energy gap among the complexes analyzed in the literature. As a result, a soft molecule has low gap energy, is more polarizable, has high chemical reactivity, and has a low level of kinetic stability. The attachment of TPA to the studied complex has given it a high IP (5.661 eV) and a high electron donating capability (ω^−^), which is 8.965 eV, as indicated in Table 2.

### Absorption spectra

**Fig. 3:**
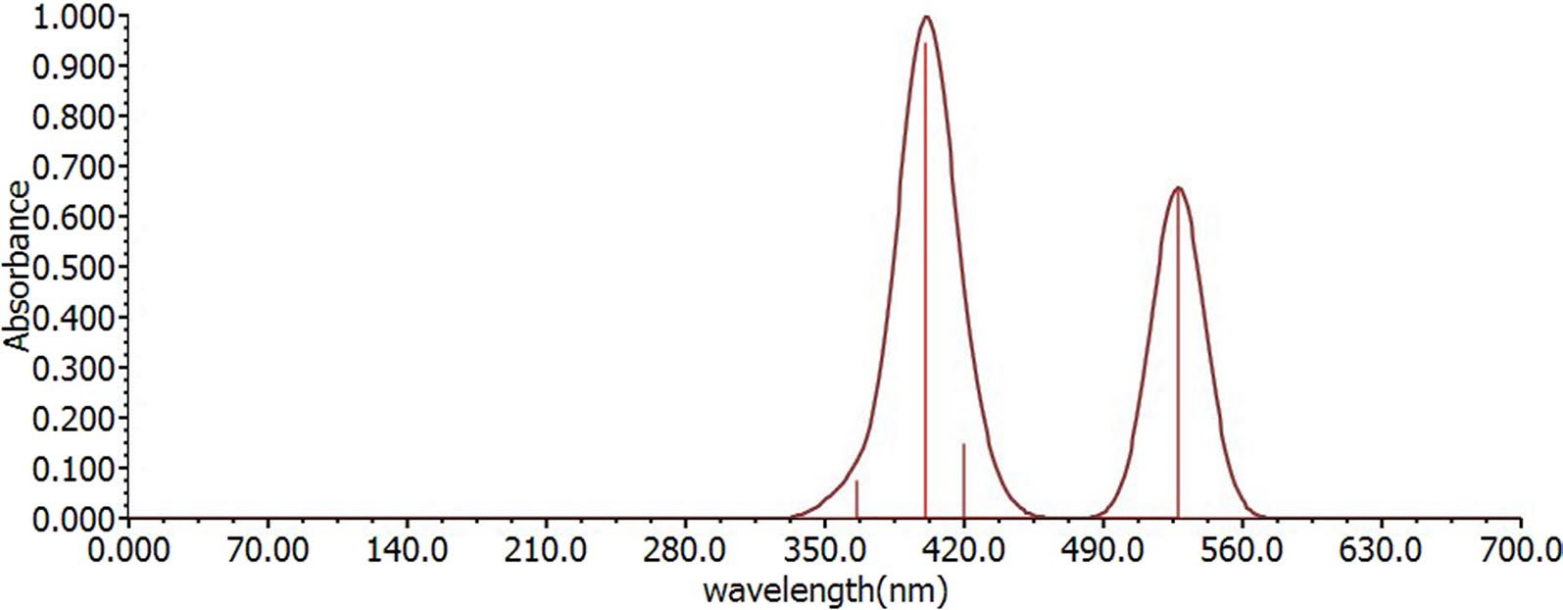
The simulated UV–Vis absorption spectra of the investigated compound

The complex's absorption characteristics have been established using the idealized ground state geometry. To identify the absorption spectra of the complex under study, PCM in CH_2_Cl_2_ medium was used in conjunction with the theoretical methods. Table 3 gathers experimental values for complex transition behavior, relevant energies/wavelengths, oscillator strength, dominating orbital excitations with configuration interaction (CI) coefficients, and their assignments from the literature [18]. Figure 3 depicts the corresponding simulated UV–Visible absorption spectra of the examined chemical using the GAMESS software. UV–Visible absorption spectrum of the studied complex is shown below (Fig. 3). Combining MLCT, XLCT, and LLCT, the H-3 to L and H to L + 2 excitations are assigned to the studied complex’s absorption band. The compounds under examination have a reduced energy absorption band of 400 nm. When EDG TPA substituents are added to the 2, 2′-bipyridine ligand (shorter wavelength), the absorption band moves to the blue.

**Table 3:** The predicted energies/wavelengths, oscillator strengths, transition character, dominant orbital excitations with CI, and their assignments for the examined complex calculated in CH_2_Cl_2_ media, as well as experimental values of complex 1 from the literature

E(eV/nm)	Oscillator strength	Transition	|CI|	Assign	$\uplambda$_exp_(nm)
2.348/528	0.3559	H$\to$L	0.703	MLCT/XLCT/LLCT	420 nm
2.793/443	0.0017	H-1$\to$L	0.699	MLCT/XLCT/LLCT	
2.948/420	0.0804	H-2$\to$L	0.695	MLCT/XLCT/LLCT	
3.096/400	0.5118	H$\to$L + 1	0.699	MLCT/XLCT/LLCT	
3.297/376	0.0000	H-3$\to$L	0.701	XLCT/LLCT	
3.381/366	0.0401	H$\to$L + 2	0.701	XLCT/LLCT	

Experimental values from [18]

### Phosphorescence spectra

**Fig. 4:**
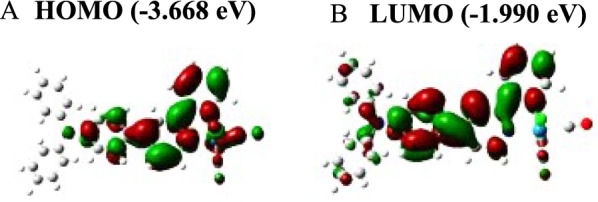
The contour plots of excited state HOMO (**A**) and LUMO (**B**) of complex

To produce the emission spectra of the complex under study, the TDDFT/B3LYP techniques with PCM in CH_2_Cl_2_ medium were applied, beginning with the optimized T_1_ structures. Table 4 shows the energy/wavelength relationships, dominating transitions with higher CI coefficients, and their assignments. In Phosphorescence, the addition of the -TPA group to complex may result in a corresponding blue shift. Furthermore, the investigated compound emits light in the visible spectrum. As a result, when a stronger EDG was added to the R positions of the 2, 2′-bipyridine ligand, the spectrum of the lowest energy emission band was blue-shifted. The contour plots of excited state HOMO and LUMO of the complex are depicted (Fig. 4).

**Table 4:** The predicted energies/wavelengths, dominating orbital excitations with large configuration interaction (CI), and complex assignments

	E (eV/nm)	Transition	|CI|	Assignment
Complex	1.5815/784	L + 3 → H	0.6790	^3^MLCT/^3^XLCT/^3^LLCT

### The complex’s chosen photovoltaic properties

#### Light harvesting efficiency

The links between the incoming photon conversion efficiency (IPCE), charge collecting efficiency (c), electron injection efficiency (Φ_inj_), and light harvesting efficiency (LHE) have been demonstrated using Eqs. (1) and (2) [22].

1 $${\text{IPCE }} = {\text{ LHE}\times} \Phi_{{{\text{inj}}}}\times \eta_{{\text{c}}}$$

2 $${\text{LHE }} = { 1} - {1}0^{ - f} .$$

where *f* is the oscillator strength that corresponds to the maximum absorption wavelength (λ_max_) in the visible or near-IR range. The absorption wavelengths were plotted against the absorptivity coefficient and oscillator strength (*f*) data to validate the transition strengths. In contrast to epsilon ('molar absorptivity,' which is determined by the molecular weight of the molecule, oscillator strengths provide a more accurate representation of the transition probability for each particular molecule. Electronic transitions in a molecule between ground states and first excited singlet states are expected to be strong because *f* values represent the degree of the transition strength and likelihood [23].

#### Excited state oxidation potential of the complex

E_ox_^Complex^, where E is the absorption energy corresponding to the complex's maximum absorption in the visible or near-IR region, and it provides the ground state oxidation potential of the complex. A considerable percentage of the energy released by the excited oxidation state of complex (E_ox_^complex^*) [22] into the TiO_2_ Conduction band is thought to come from a diffusion process [24].

3 $${\text{E}}_{{{\text{ox}}}}^{{{\text{Complex}}*}} = {\text{ E}}_{{{\text{ox}}}}^{{{\text{Complex}}}} {-} \, \Delta {\text{E}}$$

#### The diffusion coefficient D⁠π (of the π system)

As a result, the diffusion coefficient can be calculated using the Stokes' equation as shown in Eq. (4). r_complex_ is the molecular radius of the dye (Eq. 5), K_B_ is the Boltzmann constant in J/K, T is the lowest temperature in Kelvin (specified at 298.15 K), and is the viscosity of the medium [22].

4 $${\text{D}}_{\pi } = \frac{{k_{B} {\text{T}}}}{{6\pi \eta r_{complex} }}$$

#### Complex molecular radii

Suppan's equation assumes that molecular radii (r_dye_) are equal to the dyes’ respective Onsager cavity radii, a, which are calculated from the molecular volume according to Eq. (5).

5 $${\text{r}}_{{{\text{complex}}}} = {\text{ a }} = \sqrt[3]{{\frac{3M}{{4\pi \rho NA}}}}$$

where M is the molecular weight of the complex, ρ is the density of the gas (at STP), and N_A_ is the Avogadro’s number. Generally, studied complex photophysicochemical and photovoltaic characteristics were depicted in Table 5.

**Table 5:** Dye photophysicochemical and photovoltaic characteristics

	Molecular radii (r_complex_)	Diffusion coefficient (D_π_)	Oscillator strength (*f*)	Light harvesting efficiency (LHE)	Excited state oxidation potential (E_ox_^Complex*^)
Complex	2.1 $\times$ 10^–13^ nm	1.42 $\times$ 10^24^ m^2^/s	0.3559	0.559	3.313 eV

### Solvent effect on absorption and emission spectra

The polarity of various solvents varies. Different solvents produce varied excitation energies due to their polarity [25]. The PCM technique is used to evaluate solvent effects as shown in Table 6 for the complex under consideration. For complex, red shifts have been detected with decreasing solvent polarity in the lowest energy absorption and emission bands, while blue shifts observed in rising solvent polarity. When compared to the experimental technique, changes in solvents are straightforward in theoretical calculations. This is one more benefit of theoretical computations.

**Table 6:** Energy absorption and emission wavelengths of the investigated compound in solvents

Solvent	Polarity	Absorption (nm) of complex	Emission (nm) of complex
CH_2_Cl_2_	3.4	400	1195
CH_3_COCH_3_	5.4	399	1193
CH_3_OH	6.6	398	1189

### Electronic affinity (EA), ionization potential (IP) and reorganization energy (λ)

They impact how well DSSCs perform. IP and EA are regularly used to evaluate the energy hindrance for the infusion of openings and electrons from the anode into producing materials [26, 27]. Vertical and adiabatically stimulated excitations are referred to as EA (v) and EA (a), respectively (a). The electron transport revamping energy (electron), opening vehicle rearrangement energy (opening), and contrast between the electron and opening per complex were resolved involving the DFT procedure in this work and are displayed in Table 7. Vertical and adiabatically stimulated excitations are referred to as EA (v) and EA (a), respectively (a). The electron transport redesign energy (electron), opening vehicle rearrangement energy (opening), and contrast between the electron and opening per complex were resolved involving the DFT procedure in this work and are shown in Table 7. However, as demonstrated, the studied complex has a fairly small difference between electrons and holes when compared to an unsubstituted complex, which can improve charge transfer balance and further improve DSSC material efficiency. As a result, the examined chemical is better suitable for use as an emitter in DSSCs.

**Table 7:** Calculated vertical and adiabatic of EA and IP (EAv, EAa, IPv and IPa all in eV), EEP in eV, HEP in eV, λ_electron_ in eV, λ_hole_ in eV and the difference between λ_hole_ and λ_electron_ of the complex

	EA_V_	EA_a_	IP_V_	IP_a_	HEP	EEP	λ_e_	λ_h_	λ_h–e_
Complex	− 2.029	− 1.839	6.713	6.645	6.566	− 1.652	0.377	0.147	− 0.23

### The emission quantum yield in CH_2_Cl_2_ media

The conflict between radiative decay rate constant (K_r_) and non-radiative decay rate constant (K_nr_) might alter the emission quantum yield (Φ) [13].

6 $$\, \Phi {\text{ = K}}_{{\text{r}}} \tau_{{{\text{em}}}} { = }\frac{{{\text{k}}_{{\text{r}}} }}{{{\text{k}}_{{\text{r}}} {\text{ + k}}_{{{\text{nr}}}} }}$$

where, τ_em_ is the emission decay time. The large K_r_ (Eq. 7) and tiny K_nr_ (Eq. 8) are required by the preceding formula to improve the value of emission quantum yield (Φ) (Eq. 6). The K_r_ and K_nr_ can expressed as:

7 $${\text{K}}_{{\text{r}}} \, \approx \left( {\frac{{\left\langle {\Psi_{{{\text{S1}}}} \left| {{\text{H}}_{{{\text{S0}}}} } \right|\Psi_{{{\text{T1}}}} } \right\rangle^{{2}} \mu^{{_{{^{{2}} }} }}_{{_{{{\text{S1}}}} }} }}{{{(}\Delta {\text{E}}_{{\text{S1 - T1}}} {)}^{{2}} }}} \right) = \frac{{{(16}\pi^{3} {10}^{{6}} {\text{ n}}^{{3}} {\text{ E}}_{{{\text{T1}}}}^{{3}} {)}}}{{{\text{3h}}\varepsilon_{{0}} }}$$

8 $${\text{K}}_{{{\text{nr}}}} { = }\alpha {\text{e}}^{{{( - }\beta {\text{E}}_{{{\text{T1}}}} {)}}}$$

where α and β are constants, S_1_ is the electric dipole moment of transition from S_0_ to S_1_. The energy gap between S_1_ and T_1_ states is denoted by E_S1-T1_, the energy of the lowest triplet excited states for phosphorescence is denoted by E_T1_, and n, h, and ${\varepsilon }_{0}$ are the refractive index, plank's constant, and permittivity in a vacuum, respectively. As a result of the foregoing formulas, the variation of Φ can be determined qualitatively. According to the preceding equation, when E_T1_ increases, K_r_ increases and K_nr_ decreases. Table 8 summarizes the associated data. The table shows that complex has the highest E_T1_ (1.581 eV), which may raise the value of Φ. The SOC effects are mostly explained by the energy difference between the S_1_ and T_1_ states (E_S1-T1_) [28, 29]. The S_1_ and T_1_ ISC play a significant role in the phosphorescent process [30]. As ΔE_S1-T1_ grows the ISC rate decreases exponentially. The minimum E_S1-T1_ will improve an ISC rate and transition moment, perhaps increasing Kr. Table 8 shows that the studied complex has the high E_T1_ (1.581 eV), the small value of ΔE_S1-T1_ (1.174 eV), and large μ_S1_ (6.3D) As a result, it may have a higher emission quantum yield than other complexes. Among the examined complexes, the developed complex may be a viable choice for phosphorescent materials.

**Table 8:** The computed emitting energy (E_T1_/eV) and the E gap between S_1_ and T_1_ states (ΔE_S1-T1_/eV) together with the transition electric dipole moment in the S_0_ to S_1_ transition (μ_S1_/Debye)

	E_T1_ (eV)	ΔE_S1-T1_(eV)	μ_S1_(Debye)
Complex	1.5815	1.174	6.3

## Conclusion

In this study, the geometry, reactivity descriptors, light harvesting efficiency, molecular radii, diffusion coefficient, and excited oxidation potential of *fac*-[Re(I)(CO)_3_(Cl)(TPA-2, 2′-bipyridine)] were investigated using DFT and TDDFT. S_0_ and T_1_ state geometries, FMOs, reactivity descriptors, absorption and phosphorescence spectra, solvent effect, electronic affinity, ionization potential, reorganization energy, light harvesting efficiency, molecular radii, diffusion coefficient, excited oxidation potential, and emission quantum yield of the complex under investigation were specifically investigated. The addition of TPA groups to the 2, 2′-bipyridine ligand greatly modifies the electronic structures and photophysical properties such as absorption and emission spectra, charge infusion and move capacities, and emission quantum yield, according to the calculated results. The lowest-energy absorption and emission bands of this complex redden when the solvent polarity decreases, according to the solvent effect on absorption and emission spectra. Based on the results of EA, IP, and reorganization energy, we may also conclude that this complex can be used as an electron transporting material. The chosen photovoltaic properties of the complexes, such as light harvesting efficiency, molecular radii, diffusion coefficient, and excited oxidation potential, indicate the preferred complex in the use of solar cells. Furthermore, the investigated complex has the smallest electron-to-hole disparity of the complexes, which improves the device performance of DSSCs even further. The compound under investigation could have a higher quantum yield. As a result, complex is a preferable choice for usage as an emitter in DSSCs. Finally, theoretical study can afford suitable details for the intention and synthesis of novel, high-efficiency DSSC materials. Because of the TPA, a chemical that transmits holes, this combination has extraordinary light properties.

## Data Availability

The data sets used and analyzed during the current study are available from the corresponding author on reasonable request. We have presented all data in the form of Tables and Figures in the manuscript.

## Abbreviations

DFT: Density functional theory
DSSCs: Dye-sensitized solar cells
EA: Electron affinity
EDG: Electron donating group
EN: Electronegativity
EWG: Electron withdrawing group
FMOs: Frontier molecular orbitals
HOMO: Highest occupied molecular orbital
IP: Ionization potential
LANL2DZ: Los Alamos national laboratory 2 Double zeta
LHE: Light harvesting efficiency
LLCT: Ligand to ligand charge transfer
LUMO: Lowest unoccupied molecular orbital
MEP: Molecular electrostatic potential
MLCT: Metal to ligand charge transfer
N: Nucleophilicity index
PCM: Polarizable continuum model
TD-DFT: Time-dependent density functional theory
TPA: Triphenylamine
UV: Ultraviolet
XLCT: Halide to ligand charge transfer
